# Maize WRKY Transcription Factor *ZmWRKY79* Positively Regulates Drought Tolerance through Elevating ABA Biosynthesis

**DOI:** 10.3390/ijms221810080

**Published:** 2021-09-18

**Authors:** Faiza Gulzar, Jingye Fu, Chenying Zhu, Jie Yan, Xinglin Li, Tehseen Ahmad Meraj, Qinqin Shen, Beenish Hassan, Qiang Wang

**Affiliations:** 1 State Key Laboratory of Crop Gene Exploration and Utilization in Southwest China, Chengdu 611130, China; faeezagulzar@gmail.com (F.G.); cyzhu0821@163.com (C.Z.); yanjie0919@163.com (J.Y.); xinglinli0613@163.com (X.L.); tehseenahmad55@hotmail.com (T.A.M.); shenqin2016@sicau.edu.cn (Q.S.); 2 College of Agronomy, Sichuan Agricultural University, Chengdu 611130, China; 3 Rice Research Institute, Sichuan Agricultural University, Chengdu 611130, China; beenishhassan2610@gmail.com

**Keywords:** drought, maize, ABA, transcription factor, WRKY

## Abstract

Drought stress causes heavy damages to crop growth and productivity under global climatic changes. Transcription factors have been extensively studied in many crops to play important roles in plant growth and defense. However, there is a scarcity of studies regarding WRKY transcription factors regulating drought responses in maize crops. Previously, *ZmWRKY79* was identified as the regulator of maize phytoalexin biosynthesis with inducible expression under different elicitation. Here, we elucidated the function of *ZmWRKY79* in drought stress through regulating ABA biosynthesis. The overexpression of *ZmWRKY79* in Arabidopsis improved the survival rate under drought stress, which was accompanied by more lateral roots, lower stomatal aperture, and water loss. ROS scavenging was also boosted by *ZmWRKY79* to result in less H_2_O_2_ and MDA accumulation and increased antioxidant enzyme activities. Further analysis detected more ABA production in *ZmWRKY79* overexpression lines under drought stress, which was consistent with up-regulated ABA biosynthetic gene expression by RNA-seq analysis. *ZmWRKY79* was observed to target *ZmAAO3* genes in maize protoplast through acting on the specific W-boxes of the corresponding gene promoters. Virus-induced gene silencing of *ZmWRKY79* in maize resulted in compromised drought tolerance with more H_2_O_2_ accumulation and weaker root system architecture. Together, this study substantiates the role of *ZmWRKY79* in the drought-tolerance mechanism through regulating ABA biosynthesis, suggesting its broad functions not only as the regulator in phytoalexin biosynthesis against pathogen infection but also playing the positive role in abiotic stress response, which provides a WRKY candidate gene to improve drought tolerance for maize and other crop plants.

## 1. Introduction

Maize (*Zea mays* L.) is a major food and fodder crop that effectively contributes to the economic values of countries worldwide. Globally, the damage of maize crops by climatic changes has emerged as a big challenge and detrimental factor of food security [1]. Among abiotic stresses, drought has threatened and severely encumbered maize productivity in Asia, Africa, and the American continents. Long-term waterless conditions always contest crops’ survival and shift crops from growth to defense strategies. These survival attempts mostly reduce grain or biomass yield [2]. In order to cope with drought stress for sustainable maize productivity, several breeding programs are adopted, such as crop genetic engineering through the overexpression or mutation of drought-responsive genes for developing drought-tolerant transgenic crops [3,4]. In this respect, there is an urgent need to study the underlying mechanisms involving physiological responses and molecular pathways that mediate substantial stress responses.

Plants possess innate immune systems and deploy changes when encountering stress. In this case, plants sense stress signals and undergo transcriptional reprogramming to induce physiological and biochemical changes [5]. Especially, plants go for the scavenging of reactive oxygen species (ROS), osmolytes accumulation with strengthening root growth, and the closing of stomata to reduce transpiration rate [6]. Abscisic acid (ABA) is related to stomatal movement, and plants intend to optimize the level of ABA depending on the stress severity [7]. The biosynthesis of ABA is regulated by some key regulatory enzyme genes such as *NCED3* and *AAO3* [8]. The accumulation of ABA further induces the signaling responses against drought stress [9,10]. In the past years, the drought-induced response of plant cells has been extensively studied but still needs research to resolve the complex wiring of signaling networks.

Plant transcription factors (TFs) play an indispensable role in mediating stress responsiveness through signaling networks [11]. Therefore, several TF families have been stated as drought-responsive in maize crops by expressing positive or negative regulations such as ERF, MYB, WRKY, NAC, and DREBTFs [12,13,14,15]. WRKY TFs possess one or two conserved DNA binding domains with the WRKYGQK motif and the zinc-finger motif and regulate gene expression through acting on the W-box (T)(T)TGAC(C/T) *cis*-element at the promoter regions of targeted genes [16,17]. In the past few years, the role of WRKY TFs has been investigated widely in plant stress responses as well as plant growth and developmental processes [18], such as seed dormancy, flowering, and leaf senescence [19,20,21]. Currently, many WRKY genes also display a striking role in abiotic stress responses. In the Arabidopsis plant, ABO3 and AtWRKY40 facilitate ABA-induced drought stress tolerance [22]. Similarly, AtWRKY11 and AtWRKY17 affect resistance to abiotic stresses, especially salt stress [23]. In soybean, GmWRKY27 interacts with GmMYB174 to repress *GmNAC29* expression that negatively controls drought tolerance [13]. In *Pyrus betulaefolia*, PbrWRKY53 enhances drought resistance through stimulating the production of vitamin C by increasing *PbrNCED1* gene expression [24]. In Arabidopsis, AtWRKY46 and AtWRKY53 work together for negative drought regulation by assisting stomatal movement in guard cells [19]. In maize, two WRKY TFs ZmWRKY40 and ZmWRY106 have been reported as drought-responsive genes [25,26].

Our research group has characterized the function of *ZmWRKY79* in regulating maize phytoalexin biosynthesis and noticed that the gene expression of *ZmWRKY79* was enhanced under drought stress [27]. Here, we overexpressed *ZmWRKY79* in Arabidopsis for advanced investigation of its physiological and molecular mechanism under drought stress. We found that *ZmWRKY79* overexpression elevated drought tolerance in transgenic Arabidopsis plants by promoting stomatal closure through acting on the ABA biosynthetic gene.

## 2. Results

### 2.1. ZmWRKY79 Exhibited Inducible Gene Expression under Drought Stress and ABA Treatment

**Figure 1:**
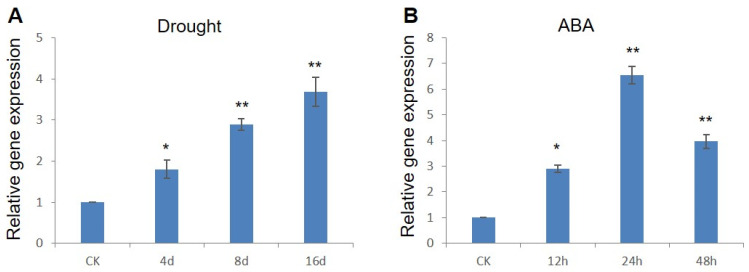
Inducible expression of *ZmWRKY79* under drought stress and ABA treatment. qRT-PCR analysis of *ZmWRKY79* gene expression in maize seedling under natural drought stress (**A**) or ABA treatment (**B**). *Ef1α* was used as the endogenous control gene, and the expression level was normalized to the untreated control (CK). Asterisks indicate significant difference compared to CK (Student’s *t*-test, * *p* < 0.05, ** *p* < 0.01). Error bars indicate SE (n = 3).

Previously, we characterized the role of the *ZmWRKY79* gene in maize terpenoid phytoalexins biosynthesis [27]. We noticed that the expression of *ZmWRKY79* was enhanced under salt and drought stresses simulated by PEG treatment, suggesting its possible involvement in abiotic stress responses [27]. To explore this potential function, gene expression of *ZmWRKY79* was analyzed in maize seedling under natural drought treatment, and significant accumulation of *ZmWRKY79* transcript was observed at 4, 8, and 16 d post treatment (Figure 1A). ABA plays a critical role in drought stress response [28]. We further analyzed *ZmWRKY79* gene expression with ABA treatment, which also significantly upregulated *ZmWRKY79* expression. A quick response of *ZmWRKY79* to ABA treatment was noted after 12 h, while the maximum transcript level was detected at 24 h (Figure 1B). These results signaled the involvement of *ZmWRKY79* in drought tolerance through ABA-dependent manner.

### 2.2. Overexpression of ZmWRKY79 Enhanced Drought Tolerance in Arabidopsis

**Figure 2:**
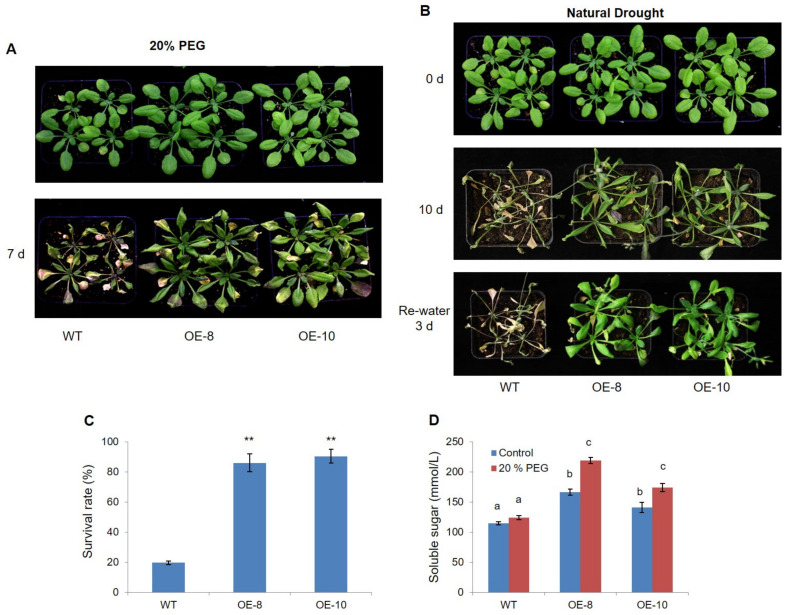
Overexpression of *ZmWRKY79* enhanced drought tolerance in Arabidopsis. (**A**) *ZmWRKY79*-OEs (OE-8 and OE-10) and WT Arabidopsis plants were treated with 20% PEG6000 treatment for 7 d. (**B**) *ZmWRKY79*-OEs (OE-8 and OE-10) and WT Arabidopsis plants were treated for natural drought for 10 d and rewatered for 3 d. (**C**) The survival rates of *ZmWRKY79*-OEs and WT seedlings with natural drought and rewatering. (**D**) Soluble sugar contents of *ZmWRKY79*-OEs and WT seedlings with 20% PEG6000 treatment. Asterisks indicate significant difference compared to WT (Student’s *t*-test, ** *p* < 0.01). Different lowercase letters indicate significant difference (LSD test, *p* < 0.05). Error bars indicate SE (n = 3).

To confirm its function in drought stress response, *ZmWRKY79* was overexpressed in Arabidopsis, and two transgenic lines (OE-8 and 10) were used for PEG6000 and natural drought treatment. Under both conditions, *ZmWRKY79* overexpression lines (*ZmWRKY79*-OEs) exhibited higher survival rates with improved growth, while the WT seedlings desiccated more rapidly than *ZmWRKY79* transgenic lines (Figure 2A–C). For natural drought treatment, the survival rate of *ZmWRKY79*-OEs was over 90%, which was considerably higher than WT plants (≈20%, Figure 2C). Additionally, the soluble sugar content of the *ZmWRKY79*-OEs was also significantly higher than that of WT plants (Figure 2D). These data indicate that the overexpression of *ZmWRKY79* increases drought tolerance.

### 2.3. ZmWRKY79 Overexpression Decreased ROS Accumulation through Elevating Antioxidant Enzyme Activity under Drought Stress

**Figure 3:**
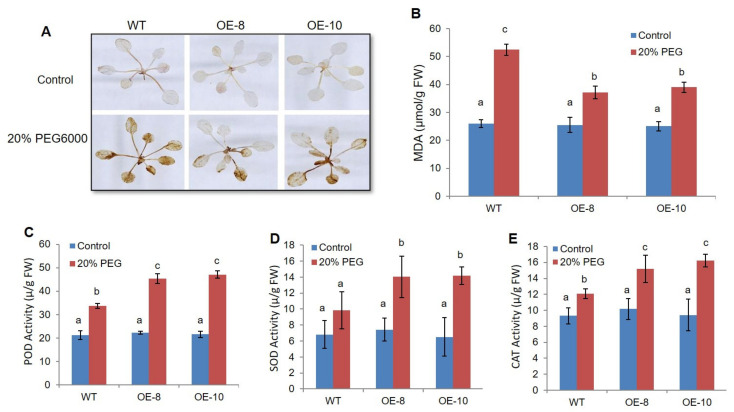
*ZmWRKY79* overexpression decreased ROS accumulation through elevated antioxidant enzyme activity under drought stress. (**A**) H_2_O_2_ accumulation by DAB staining in *ZmWRKY79*-OEs (OE-8 and OE-10) and WT plants with 20% PEG 6000 treatment. (**B**–**E**) MDA content (**B**) and antioxidant enzyme activity for CAT (**C**), SOD (**D**), and POD (**E**) in *ZmWRKY79*-OEs (OE-8 and OE-10) and WT plants with 20% PEG 6000 treatment. Different lowercase letters indicate significant difference (LSD test, *p* < 0.05). Error bars indicate SE (n = 3).

The overproduction of ROS causes oxidative injury in plants under drought stress. To assess the role of *ZmWRKY79* in response to oxidative pressure, ROS accumulation was investigated under drought stress in *ZmWRKY79*-OEs and WT Arabidopsis plants through DAB staining. After PEG treatment for 7 d, *ZmWRKY79*-Oes accumulated less H_2_O_2_ than WT, as most of the WT plants turned brown and necrotic. Meanwhile, no dissimilarity was witnessed between *ZmWRKY79* transgenic lines and WT seedlings under normal growth condition (Figure 3A). In addition, drought stress also causes membrane lipids damage through excessive ROS accumulation [4]. We also investigated membrane lipid damage in both *ZmWRKY79*-OEs and WT plants under drought stress. The results showed that there was less MDA production in *ZmWRKY79*-OEs as compared to WT plants (Figure 3B), indicating less membrane damage by *ZmWRKY79* overexpression. These results suggested that *ZmWRKY79* might be involved in ROS homeostasis and the regulation of cellular redox to control the oxidative injury triggered by drought stress.

Plants can protect themselves from oxidative damage through the ROS-scavenging system in which antioxidant enzymes such as SOD, CAT, and POD act as ROS scavengers to protect plants [6]. Here, we measured the enzyme activities such as POD, CAT, and SOD in *ZmWRKY79*-OEs and WT plants under both control and drought conditions. The results revealed that the antioxidant enzyme activities of *ZmWRKY79*-OEs were significantly higher than WT seedlings under drought conditions. In control conditions, no variations were recorded in antioxidant enzyme activities between *ZmWRKY79*-OEs and WT plants (Figure 3C–E). These results further supported that the overexpression of *ZmWRKY79* improves drought tolerance by boosting the antioxidant enzyme activities in transgenic plants.

### 2.4. ZmWRKY79 Overexpression Promoted Stomatal Closure by Enhancing ABA Synthesis under Drought Stress

**Figure 4:**
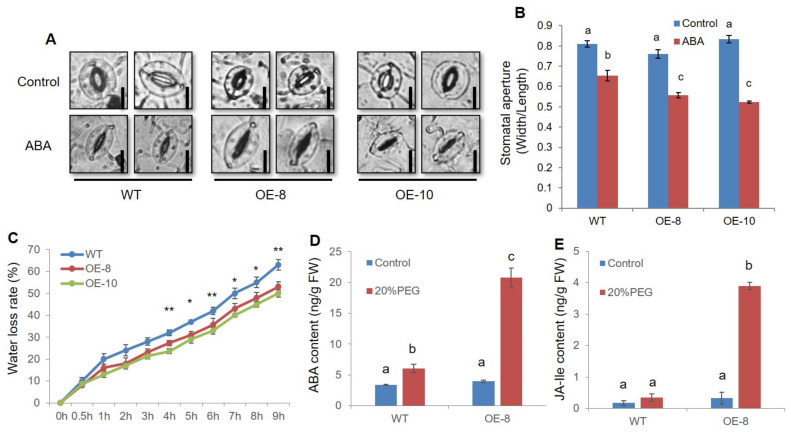
*ZmWRKY79* overexpression promoted stomatal closure and ABA synthesis. (**A**) Stomata of *ZmWRKY79*-OEs (OE-8 and OE-10) and WT Arabidopsis plants with 5 μM ABA treatment. The untreated samples were used as the control. Bars mean 20 μm. (**B**) Stomatal aperture of OE-8, OE-10, and WT with or without ABA treatment. (**C**) Water loss rates of OE-8, OE-10, and WT-detached leaves at room temperature. (**D**–**E**) Accumulation of ABA (**D**) and JA-Ile (**E**) in OE-8 and WT with 20% PEG6000 treatment. Asterisks indicate significant difference compared to WT (Student’s *t*-test, * *p* < 0.05, ** *p* < 0.01). Different lowercase letters indicate significant difference (LSD test, *p* < 0.05). Error bars indicate SE (n = 3).

Stomatal movement is regulated by ABA signal in response to drought stress, which controls transpiration and water loss [29]. After ABA treatment, more drastic stomatal closure was observed for *ZmWRKY79*-OEs, which was demonstrated by the lower stomatal aperture in *ZmWRKY79*-OE leaves (Figure 4A,B). We further analyzed water loss for *ZmWRKY79*-OE and WT plants. Significant lower water loss rates were detected for two *ZmWRKY79*-OE lines (Figure 4C), which is consistent with the lower stomatal aperture preventing water loss by *ZmWRKY79* overexpression. Moreover, ABA content was measured under drought stress simulated by 20% PEG6000 treatment. *ZmWRKY79*-OE seedlings accumulated almost three times higher ABA content than WT seedlings after PEG6000 treatment (Figure 4D). In addition to ABA content, JA-Ile, the active form of JA, was also detected with ≈4 times higher in *ZmWRKY79*-OE lines than WT plants (Figure 4E). These results indicated that *ZmWRKY79* upregulates ABA synthesis under drought stress, thereby promoting stomatal closure and conferring lower water loss in transgenic plants.

### 2.5. ZmWRKY79 Transgenic Lines Had Efficient Root Systems under Drought Stress

**Figure 5:**
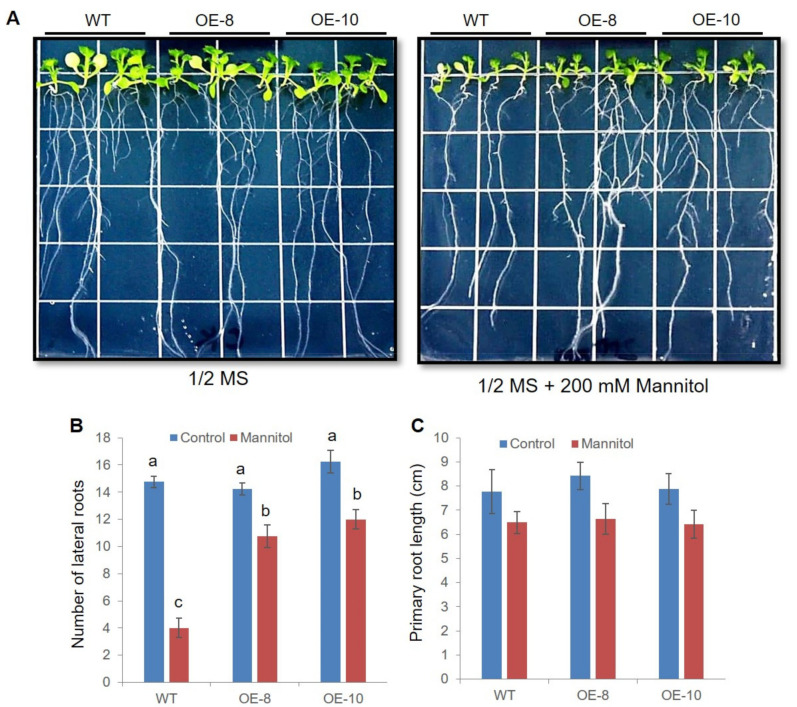
*ZmWRKY79* overexpression improved later root growth under mannitol treatment. (**A**) Root growth of *ZmWRKY79*-OEs (OE-8 and OE-10) and WT Arabidopsis seedlings under normal growth condition (1/2 MS) or 200 mM mannitol treatment. (B, C) Lateral root number (**B**) and primary root length (**C**) of OE-8, OE-10, and WT under mannitol treatment. Different lowercase letters indicate significant difference (LSD test, *p* < 0.05). Error bars indicate SE (n = 3).

Plants usually elevate root growth in response to drought stress for more water intake. We analyzed root growth regulated by *ZmWRKY79* in OE lines. Under normal growth condition, no obvious difference was observed in the growth of WT and *ZmWRKY79*-OEs on the control MS medium (Figure 5). In contrast, under mannitol treatment, the *ZmWRKY79*-OEs showed a substantial increase in the number of lateral roots, while the primary root length did not differ significantly (Figure 5B,C). The *ZmWRKY79*-OEs had more than two times lateral roots than WT (Figure 5B), indicating stronger drought tolerance.

### 2.6. Transcriptome Profiling of ZmWRKY79 Target Genes in Response to Drought Stress

**Figure 6:**
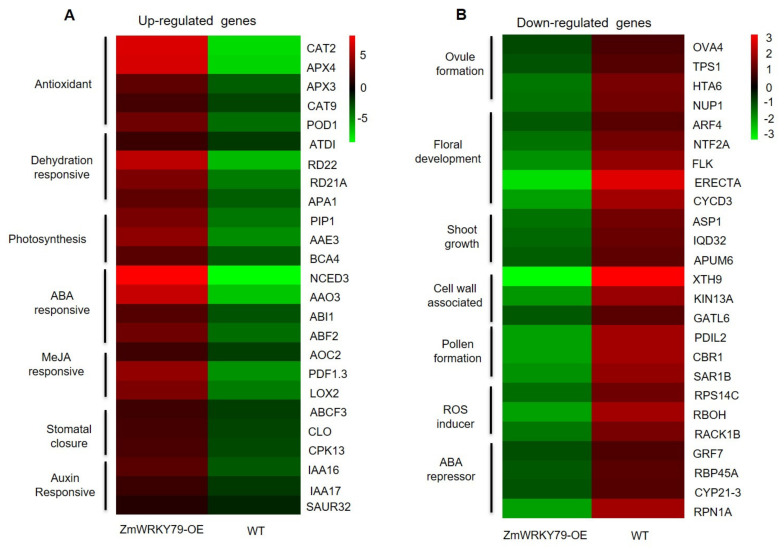
Key differentially expressed genes (DEGs) in WT and *ZmWRKY79*-OE plants under drought stress. Up-regulated (**A**) and down-regulated genes (**B**) in WT and *ZmWRKY79*-OE plants under drought stress simulated by 20% PEG6000 treatment for 24 h. The color scale indicates the mean fold-change of three biological replicates.

To explore the underlying molecular mechanism of elevated drought tolerance by *ZmWRKY79* overexpression, RNA-seq analysis was conducted using the transgenic and WT Arabidopsis plant with drought stress treatment. In total, 157 genes were up-regulated and 340 genes were down-regulated in *ZmWRKY79*-OE plants as compared to WT (Figure S1). Up-regulated genes were mostly related to antioxidant enzyme activity, dehydration responses, transporter activity, and plant hormone signaling, which specifies the stronger activity of drought responses in *ZmWRKY79*-OE plants than WT (Figure 6A and Figure S2). On the other hand, most of the down-regulated genes were enriched into growth and development-related processes (Figure 6B and Figure S2), indicating repressed growth in *ZmWRKY79* overexpression plants under drought stress, which should be the strategy to deal with the acute environment.

According to the KEGG pathway enrichment, the plant hormone signaling pathways were significantly enriched in *ZmWRKY79*-OE Arabidopsis under drought conditions (Figure S3). A number of up-regulated genes were related to ABA biosynthesis and signaling, such as ABA biosynthetic genes *NCED3* (AT3G14440), *AAO3* (AT2G27150), and the ABA signaling gene *ABI5* (AT2G36270). Considering the positive role of ABA in regulating stomatal closure, the enhanced stomatal closure in *ZmWRKY79*-OE plants might be a result of elevated expression of ABA-related genes. In addition, numerous JA-related genes, including *AOC2* (AT3G25770) and *OPR2* (AT1G76690), were up-regulated in *ZmWRKY79*-OEs (Figure 6A, Supplementary Dataset 1–3), which is consistent with the enhanced JA level in transgenic lines compared with WT (Figure 4E).

Two essential genes against dehydration including *RD22* (AT5G25610) and *RD29A* (AT5G52310) and several antioxidant enzyme-related genes such as *AtCAT3* (AT1G20620) and *AtPOD1* (AT1G67960) were also identified (Supplementary Dataset 1). All these genes showed up-regulation in *ZmWRKY79*-OE plants under drought stress, which explains the higher sugar content and higher antioxidant enzyme activity under drought stress detected in *ZmWRKY79*-OE seedlings (Figure 2D and Figure 3C–E). Moreover, some dehydration responsive genes, stomatal movement related genes, as well as some MAPK genes also exhibited up-regulation in *ZmWRKY79*-OE plants under drought stress, suggesting the broad effects of *ZmWRKY79* in drought stress response.

**Figure 7:**
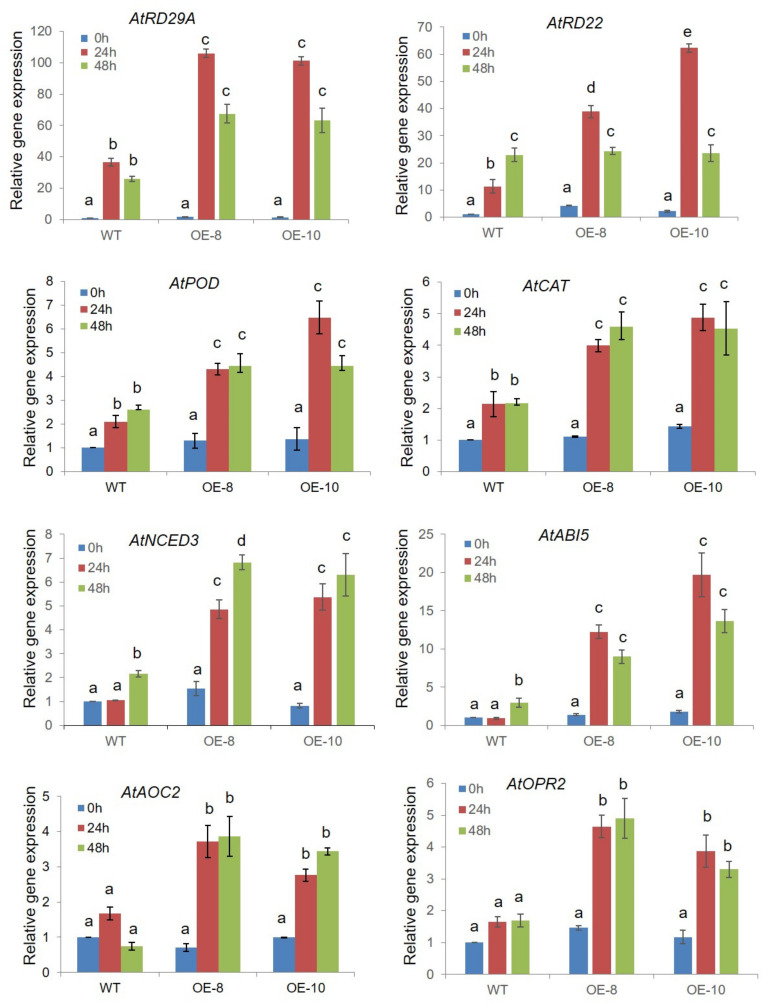
Gene expression validation of *ZmWRKY79* regulated genes. qRT-PCR analysis of *ZmWRKY79* regulated genes in OE plants (OE-8 and OE-10) and WT plants with 20% PEG6000 treatment for 0, 24, and 48 h. *Atactin* was used as the endogenous control gene, and the expression level was normalized to the untreated WT (0 h). Different lowercase letters indicate significant difference (LSD test, *p* < 0.05). Error bars indicate SD (n = 3).

To confirm the RNA-seq results, eight DEGs from different categories were picked for qRT-PCR analysis (Figure 7). The expression levels of drought-related genes *RD29A* and *RD22* were increased in *ZmWRKY79*-OE lines under drought conditions. Similarly, the expression of ABA and JA related genes, as well as antioxidant enzyme-related genes, were also significantly up-regulated in *ZmWRKY79*-OEs in response to drought treatment. Taken together, these results strongly demonstrated that *ZmWRKY79* mediated drought tolerance by affecting ABA biosynthesis and signaling.

### 2.7. ZmWRKY79 Stimulated Expression of ABA Biosynthetic Gene AAO3

**Figure 8:**
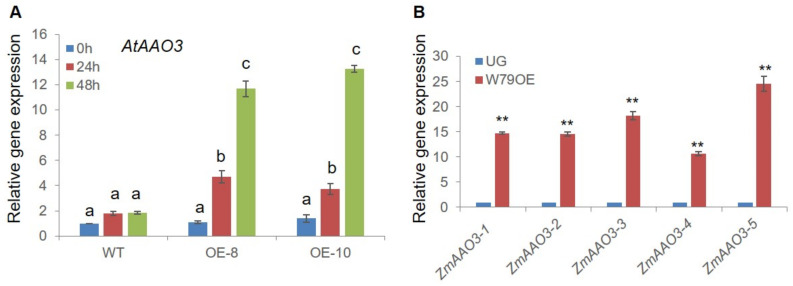
*ZmWRKY79* promoted the expression of the ABA biosynthetic gene *AAO3*. (**A**) qRT-PCR analysis of *AtAAO3* gene expression in *ZmWRKY79*-OEs (OE-8 and OE-10) and WT Arabidopsis plants with 20% PEG6000 treatment for 0, 24, and 48 h. *Atactin* was used as the endogenous control gene, and the expression level was normalized to the untreated WT (0 h). Different lowercase letters indicate significant differences (LSD test, *p* < 0.05). (**B**) qRT-PCR analysis of five *ZmAAO3* homologous genes in maize protoplast with transient overexpression of *ZmWRKY79*. UG means the empty vector control. *Ef1a* was used as the endogenous control. Asterisks indicate significant difference compared to the control (UG) (Student’s *t*-test, ** *p* < 0.01). Error bars indicate SE (n = 3).

*AtAAO3* and *AtNCED3* have been identified as being involved in ABA biosynthesis, and their high expression resulted in boosted endogenous ABA levels and stomatal closure [30,31]. Exposure to water stress slightly triggered the regulation of *AtNCED3* and *AtAAO3* in the WT, but enhanced expression was detected in the *ZmWRKY79*-OE lines (Figure 7 and Figure 8A), suggesting that *ZmWRKY79* might influence ABA biosynthesis directly. To further uncover the regulation of ABA biosynthetic genes, *ZmWRKY79* was transiently overexpressed in maize protoplast, and ABA biosynthetic genes were analyzed. In agreement with the data obtained in the *ZmWRKY79*-OE Arabidopsis seedlings, the transient overexpression of *ZmWRKY79* in maize protoplast also promoted the expression of maize homology genes of *AtAAO3*. All five *ZmAAO3* genes were up-regulated in maize protoplast with *ZmWRKY79* transient overexpression (Figure 8B), while the maize homology genes of *AtNCED3* were not affected significantly (Figure S4), indicating that *ZmWRKY79* might not directly target *ZmNCED3.*

### 2.8. ZmWRKY79 Activated Promoters of ZmAAO3 Genes

**Figure 9:**
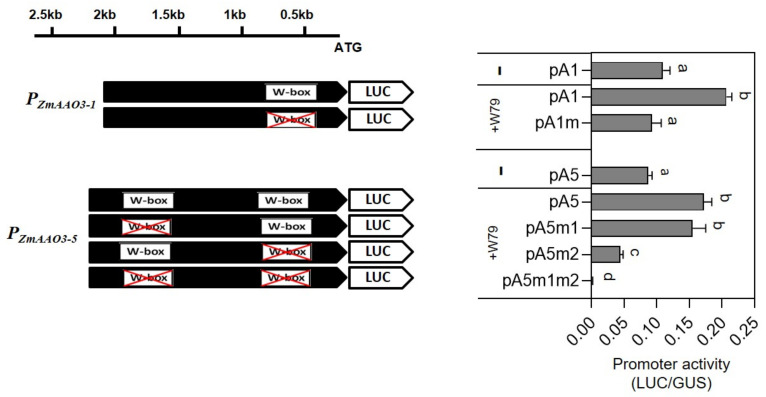
*ZmWRKY79* activated the promoter of the *ZmAAO3* genes. Promoter-LUC assay of *ZmAAO3-1* and *ZmAAO3-5* activated by *ZmWRKY79* in maize protoplast. *ZmAAO3* promoters (pA1 designate *P_ZmAAO3-1_* and pA5 designate *P_ZmAAO3-5_*) were inserted into the fusion construct to drive the luciferase (LUC) reporter gene. Promoter activities with (+) or without (−) of the effector *ZmWRKY79* (W79) were shown by the LUC/GUS ratio (relative LUC activity). Mutations (M) of W-boxes are indicated with Χ. Different lowercase letters indicate significant difference (LSD test, *p* < 0.05). Error bars indicate SE (n = 4).

To characterize the regulatory mechanism of *ZmWRKY79*, the promoters of two maize *AAO3* genes (*ZmAAO3-1* and *ZmAAO3-5*) were cloned and analyzed through the promoter–reporter system in maize protoplast. The co-transfection of *ZmWRKY79* significantly elevated the promoter activities of *ZmAAO3* genes (Figure 9), suggesting direct activation on promoters by *ZmWRKY79*, which is consistent with the up-regulated *ZmAAO3* gene expression in maize protoplast with *ZmWRKY79* transient overexpression.

WRKY TFs bind to the W-box or W-box-like elements (WLE) to regulate promoter activity and gene expression. Further analysis identified one W-box element in the *ZmAAO3-1* promoter and two W-boxes in the *ZmAAO3-5* promoter. Mutation of the W-box in the *ZmAAO3-1* promoter situated at −530 to −536 bp drastically compromised the activation of *ZmWRKY79*, indicating that *ZmWRKY79* activated ZmAAO3-1 gene expression through acting on this W-box. On the other hand, mutation of the first W-box (−1817 to −1823 bp) in the *ZmAAO3-5* promoter did not change the activity significantly. Further mutation in the second W-box at −580 to −586 bp from ATG considerably reduced the activation of the promoter. Moreover, extreme activity loss was noted on the mutation of both W-boxes of the *AAO3-5* promoter (Figure 8). The results specified that in the case of the *AAO3-5* promoter, both W-boxes acted, but the second one (−580 to −586 bp) played the main role in activation by *ZmWRKY79*.

### 2.9. Silencing of ZmWRKY79 Attenuated Drought Resistance in Maize

**Figure 10:**
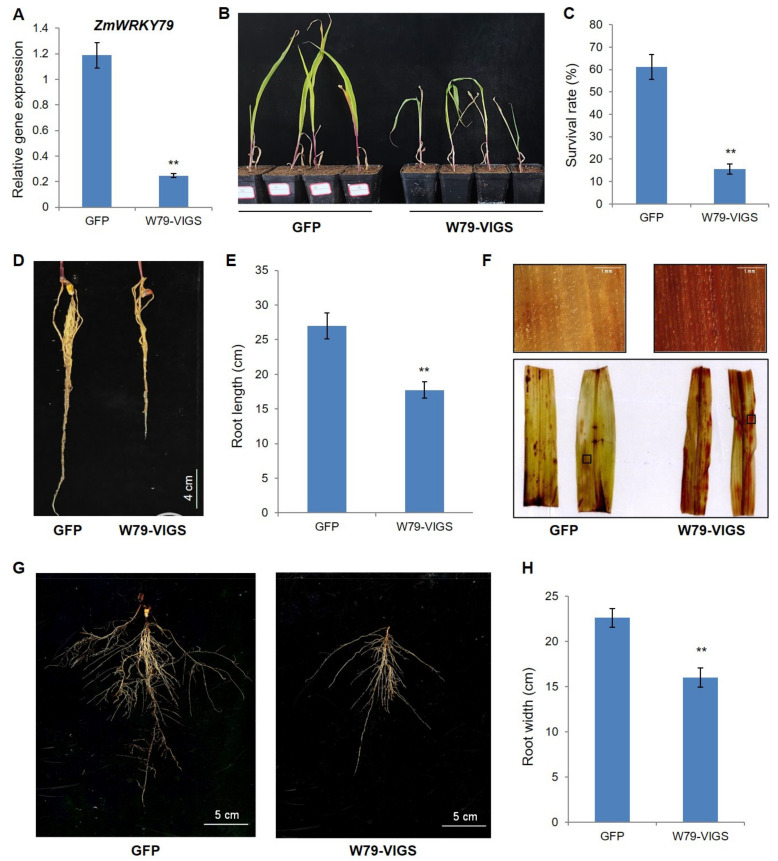
Silencing of *ZmWRKY79* reduced drought resistance in maize. (**A**) Gene expression of *ZmWRKY79* in maize seedlings with VIGS. GFP was used as the control. (**B**) Maize seedlings with VIGS of *ZmWRKY79* under drought stress as compared to control plants (GFP). (**C**) Survival rate of maize seedlings with VIGS of *ZmWRKY79* or the control plants (GFP) under drought stress. (**D**) Roots of maize seedlings with or without VIGS of *ZmWRKY79* under drought stress. (**E**) Root length of maize seedlings with or without VIGS of *ZmWRKY79* under drought stress. (**F**) H_2_O_2_ accumulation by DAB staining in maize leaves with or without VIGS of *ZmWRKY79* under drought stress. (**G**) Representative picture of root width of maize seedlings with or without VIGS of *ZmWRKY79* under drought stress. (**H**) Root width of maize seedlings with or without VIGS of *ZmWRKY79* under drought stress. Asterisks indicate significant difference compared to the control (GFP) (Student’s *t*-test, ** *p* < 0.01). Error bars indicate SE (n = 3).

To further investigate the biological function of *ZmWRKY79* in maize drought resistance, virus-induced gene silencing (VIGS) was subsequently employed to knock down the expression of *ZmWRKY79* in maize. qRT-PCR analysis showed that the transcripts of *ZmWRKY79* were significantly decreased in *ZmWRKY79*-silenced maize plants compared to the control (GFP-treated maize) (Figure 10A), suggesting the successful silencing of *ZmWRKY79*. The growth of *ZmWRKY79*-silenced plants (W79-VIGS) showed no obvious difference with control plants (Figure S5). Then, the drought tolerance of W79-VIGS plants was examined. After natural drought treatment for 12 d, the *ZmWRKY79*-silenced plants exhibited severe wilting, while the GFP-VIGS plants maintained relatively better growth (Figure 10B), indicating the positive regulation of *ZmWRKY79* in maize drought resistance. The survival rate of W79-VIGS plants was also significantly lower than the control after rewatering for 3 d (Figure 10C). Based on the enhanced lateral root growth observed in *ZmWRKY79*-OE Arabidopsis, we compared the root architecture of W79-VIGS plants and the control plants after drought treatment. As shown in Figure 10D,G, the root growth of W79-VIGS plants was apparently weaker than the control plants, with significantly reduced root length and width (Figure 10E,H). Further DAB staining revealed more H_2_O_2_ accumulation in W79-VIGS leaves (Figure 10F), which is consistent with the results observed in transgenic Arabidopsis, confirming the role of *ZmWRKY79* in activating the ROS-scavenging system under drought stress. Moreover, we detected the expression of *ZmAAO3-1* and *ZmAAO3-5* in silenced maize plants under normal and drought conditions. The results showed that the expression of *AAO3* genes was increased in GFP plants with drought treatment, while the induction was compromised in W79-VIGS plants (Figure S6). Combined with the reduced survival rates and compromised growth of W79-VIGS plants, these results strongly indicated that the attenuated drought tolerance in W79-VIGS maize is at least partially dependent on the down-regulated ABA biosynthestic genes.

## 3. Discussion

Maize is the main food and commercial crop and has a central role in basic biological research. Information about the function of WRKY TFs in abiotic stresses is inadequate in maize. Previously, we characterized the regulatory function of *ZmWRKY79* in phytoalexin biosynthesis and disease resistance [27]. We noticed that a few abiotic-related factors also induced *ZmWRKY79* gene expression [27]. Here, we identified the positive role of *ZmWRKY79* in drought stress response. The transgenic Arabidopsis plants with *ZmWRKY79* overexpression displayed a better survival rate and phenotypic morphology under drought treatment (Figure 2). Notably, more ABA production was detected in *ZmWRKY79*-OE lines than WT with drought treatment (Figure 4D). ABA is a major phytohormone playing the positive role in drought stress response to regulate a number of physiological processes including stomatal closure [30], which is essential for reduced water loss under drought conditions [32,33]. Consistent with more ABA accumulation, *ZmWRKY79*-OE lines exhibited lower stomatal aperture and water loss (Figure 4A–C), contributing to drought tolerance in these transgenic plants with *ZmWRKY79* overexpression. Further analysis revealed a higher gene expression of ABA biosynthesis and signaling in *ZmWRKY79*-OEs than WT under drought stress by RNA-seq and qRT-PCR analysis (Figure 6 and Figure 7), indicating that *ZmWRKY79* elevated drought tolerance by regulating ABA synthesis and signaling. This regulatory mechanism was further confirmed in maize protoplast where the transient overexpression of *ZmWRKY79* significantly promoted *ZmAAO3* gene expression through acting on W-boxes in the promoters of these genes (Figure 8 and Figure 9), implicating that *ZmWRKY79* might regulate ABA synthesis in maize to confer resistance against drought and/or other abiotic stresses. Furthermore, VIGS of *ZmWRKY79* in maize markedly enhanced the susceptibility to drought stress (Figure 10), demonstrating the positive role for *ZmWRKY79* to increase the drought tolerance in maize.

Based on the conserved WRKY domain, 125 possible WRKY TFs were identified in the maize B73 inbred line genome [34]. We performed the phylogenetic analysis of these WRKY proteins and found that *ZmWRKY79* was most closed to ZmWRKY121, with 70.08% sequence identity (Figure S7). Another two related genes are ZmWRKY38 and ZmWRKY37, with 56.14% and 38.79% sequence identity, respectively. The biological functions of ZmWRKY121 and ZmWRKY38 remain unclear, and future exploration is needed. *ZmWRKY37* was identified as a strong co-expressed gene with cold-responsive gene *ZmFAD2.1* [35]. This gene has also been detected as a differentially expressed gene under *Fusarium* ear rot infection [36], suggesting that ZmWRKY37 might also be a dual functional gene involved in both biotic stress and abiotic stress, similarly to *ZmWRKY79*, which has been identified previously as a positive regulator of maize terpenoid phytolexins in response to pathogen infection [27], while in our study, it showed great potential to enhance drought tolerance in maize.

AAO3 plays a significant role in ABA biosynthesis to catalyze the production of abscisic acid from abscisic aldehyde [37]. The previous investigation declared that *AAO3* was primarily responsible for the synthesis of ABA under drought as the *AtAAO3* knockout mutant displayed a drought-sensitive phenotype along with decreased ABA level [9,10]. ZmPFT1 has been reported to directly control the ABA biosynthetic gene *NCED3* [30], but there is no research on the direct activator of *AAO3* in maize. In this study, *ZmWRKY79* up-regulated *AtNCED3* and *AtAAO3* gene expression in Arabidopsis (Figure 7 and Figure 8A) but only acted on *ZmAAO3* in maize protoplast (Figure 8B), which might reveal the distinct stress tolerance mechanism in maize and should be explored in future research.

JA has been reported to function synergistically with ABA to promote stomatal closure and decrease the water loss under drought stress [38]. In our study, we also detected higher JA-Ile production in *ZmWRKY79*-OE lines under drought stress (Figure 4E). Consistently, *ZmWRKY79* has been found to up-regulate several JA-related genes in maize protoplast [27]. We also detected some JA biosynthesis and signaling genes up-regulated with *ZmWRKY79* overexpression in transgenic Arabidopsis plants (Figure 6 and Figure 7). Together with the enhanced stomatal closure observed in *ZmWRKY79*-OE plants (Figure 4A,B), a possible involvement of *ZmWRKY79* is implied in regulating ABA and JA mediated stomatal closure. In addition, complicated signaling cross-talk has been identified between ABA and JA to be involved in many biological processes. In maize, both ABA and JA signaling play roles in phytoalexin biosynthesis, and drought treatment also induced phytoalexin accumulation in maize roots [39,40]. In this study, enhanced ABA and JA biosynthesis by *ZmWRKY79* suggests broader functions not only acting on phytoalexin biosynthesis but also playing an important role on plant hormone (i.e., ABA and JA) signaling to be involved in more biological processes and environmental response.

In plants, primary roots and lateral roots are of extreme significance, as they are mostly participating in water and nutrient uptake and are vital organs for adapting to various environments [30]. Generally, longer roots and widespread lateral roots are positively correlated to sustained plant survival in dehydrated conditions [41]. Under stress conditions, *ZmWRKY79*-OE lines exhibited more abundant lateral roots (Figure 5), which would boost the uptake of nutrients and water, positively contributing to drought tolerance. Furthermore, drought stress triggers ROS accumulation, thereby causing membrane damage with lipid peroxidation to produce MDA [42]. MDA content is normally used to evaluate the severity of membrane lipid damage. Under drought stress, less H_2_O_2_ was detected, as well as less MDA accumulation in transgenic plants, which is consistent with higher antioxidant enzyme activities in *ZmWRKY79*-OEs (Figure 3). The overexpression of *NAC042* has been reported to increase drought tolerance in banana, which also decreased the accumulation of H_2_O_2_ and MDA [43]. Regulation of the ROS scavenging system with improved antioxidant capability should be the common mechanism to enhance drought tolerance. In this study, our results clearly indicated that *ZmWRKY79* overexpression elevated antioxidant enzyme activity and resulted in less ROS accumulation, echoing the inducible expression of *ZmWRKY79* by H_2_O_2_ treatment in previous investigation [27].

Notably, the overexpression of *ZmWRKY79* did not change the growth and morphology of transgenic Arabidopsis plants (Figure 2). Under normal growth condition, no significant difference was detected for ABA or JA production between *ZmWRKY79*-OE lines and WT plants (Figure 4D,E). Some unknown mechanisms or unidentified factors might inhibit the function of *ZmWRKY79* on promoting ABA synthesis under normal growth conditions, which might be post-translational modification or some co-factors that could be initiated by stress conditions. Such mechanisms could be the part of the fine-tuning system for the growth–defense balance and need to be investigated in the future research.

Overall, this study revealed that *ZmWRKY79* mediated drought tolerance positively depending on an increment of ABA level by triggering ABA biosynthetic genes including *AAO3* genes. This WRKY-*AAO3* module offers new valuable information related to the role and basic molecular mechanism of WRKY TFs and elaborates our knowledge about the tricky drought signaling network. Still, more research is compulsory to unravel other mechanisms related to *ZmWRKY79*-mediated drought tolerance to achieve a precise and defined molecular mechanism. In the future, it will be more valuable to assess the performance and reliability of *ZmWRKY79*-OE plants under field conditions to estimate the negative and positive influence of ABA on plant production and drought tolerance.

## 4. Materials and Methods

### 4.1. Plant Materials and Treatments

Seeds of maize (Mo17 inbred line) were grown for about 2 weeks in the soil with a photoperiod of 16 h/8 h (light/dark) at 28 °C. The same quantity of soil was used for each pot, and the soil was frequently watered before starting drought treatment. At the 3-leaf stage, natural drought was applied by stopping the water supply for 16 days. Then, the aerial tissues of the seedlings were collected for RNA extraction at various time points (0 d, 4 d, 8 d, and 16 d), and cDNA was synthesized for gene expression analysis by qRT-PCR.

### 4.2. RNA Extraction and qRT-PCR Analysis

Total RNA was extracted with the TRNzol reagents (Tiangen, Beijing) following the manufacturer’s protocol. cDNA was synthesized by the M-MLV reverse transcriptase from Takara. qRT-PCR was executed on an ABI Prism 7500 system (Applied Biosystems) using the SsoFast EvaGreen Supermix (Bio-Rad). The maize gene *Ef1a* or Arabidopsis gene *AtActin* was used as the endogenous control gene according to previous studies [44]. All experiments were conducted with at least three replicates. The primers for qRT-PCR analysis are listed in Table S1.

### 4.3. Generation of Transgenic Arabidopsis Plants

For Arabidopsis transformation, the CDS of *ZmWRKY79* was cloned from the cDNA of maize leaves and ligated into the pCAMBIA3301 vector under the control of the CaMV 35S promoter. pCAMBIA3301/*ZmWRKY79* was further transformed into *Agrobacterium tumefaciens* GV3101. Arabidopsis transformation was conducted with the floral dipping method with *Arabidopsis thaliana* Col-0. The positive transgenic plants were screened by Basta, and at least two independent transgenic plants were obtained until the T3 generation for further analysis.

### 4.4. Drought Tolerance Assessment in Transgenic Lines

Arabidopsis Col-0 (WT) and *ZmWRKY79* overexpression transgenic lines were exposed to both induced (20% PEG 6000) and natural drought (10 d of dewatering) to assess their stress tolerance capability. For natural drought, after 20–25 d of germination, the water supply of both *ZmWRKY79*-OEs and WT was stopped for 10 d, and after that, the same plants were reimbursed to normal irrigation for 3 d to check out their survival rates. In the case of induced drought, *ZmWRKY79* overexpression seedlings and WT plants were treated with 20% PEG6000 for 7 d, and then, survival rates were recorded. The leaves were also collected to determine soluble sugar content and malondialdehyde (MDA) content as well as antioxidant enzyme activity. All experiments were conducted with at least three replicates.

### 4.5. Root Growth of ZmWRKY79 Overexpression Arabidopsis

WT and *ZmWRKY79* overexpression Arabidopsis seeds were sterilized and plated on 1/2 MS solid medium at 4 °C for 3 d vernalization and transferred to 1/2 MS solid medium with or without 200 mM mannitol. Seedlings grown for 2 weeks were photographed, the root length was measured, and the number of lateral roots was counted. Each assay was performed with three biological replicates.

### 4.6. Antioxidant Enzyme Activity, DAB Staining, MDA, and Soluble Sugar Content

The antioxidant enzyme activities, including superoxide dismutase (SOD), catalase (CAT), and peroxide dismutase (POD) were measured in *ZmWRKY79* transgenic lines and WT plants using the control and drought-treated samples, as previously measured [45]. MDA content was measured by using thiodaibarbital acid (TBA) as substrates to produce thiobarbituric acid–malondialdehyde (TBAMDA), which has an absorbance at 532 nm [46]. For soluble sugar measurement, the leaf samples were extracted with 5% phenol and 98% sulfuric acid, and then, the absorbance of the filtered mixture at 485 nm was detected using a spectrophotometer. Four-week-old Arabidopsis plants after treatment of 20% PEG 6000 at for 7 d were used for 3, 3-diaminobenzidine (DAB) staining. The aerial parts were cut and submerged in the tubes filled with 1 mg/mL DAB solution (pH = 3.8). DAB dye was discarded after 1 d, and samples were washed by 95% ethanol and photographed.

### 4.7. Analysis of Endogenous ABA and JA Contents

Four-week-old *ZmWRKY79* overexpression line (OE-8) and WT Arabidopsis plants were treated with 20% PEG6000 for 48 h. Aerial tissues (≈100 mg) were harvested, frozen in liquid N_2_, and ground into fine powder for extraction with 1 mL of methanol/water/formic acid (15:4:1, V/V/V). The combined extracts were concentrated and resuspended into 100 μL 80% methanol (V/V) for further LC-MS analysis. The endogenous ABA and JA-Ile contents were detected by MetWare (http://www.metware.cn/, accessed on 15 September 2021) based on the AB SciexQTRAP 6500 LC-MS/MS platform. Three biological replicates from each line were analyzed.

### 4.8. Water Loss from Detached Leaves

To record the water loss rate, aerial parts of three-week-old Arabidopsis plants were cut and kept at room temperature with 40% humidity in culture plates. Then, leaves were weighed at specified time points to assess water loss. At least three replications were used for each sample.

### 4.9. Measurement of Stomatal Aperture

For stomatal aperture measurement, three-week-old Arabidopsis leaves were collected and dipped into a stomatal opening solution to induce stomatal opening as described previously [47]. After that, 5 μM ABA was mixed into the solution for 2 h. The stomatal activity was scrutinized under a microscope after ABA treatment from each leaf strip. Then, ImageJ software was used to measure stomatal aperture.

### 4.10. Preparation of Maize Leaf Protoplast along with Plasmid Transfection

The polyethylene glycol/calcium-mediated transformation technique was used to perform this experiment [27,48]. First, 10-day-old maize etiolated seedlings (Mo17) were selected for protoplast formation. Firstly, the second leaf of each plant was cut into 1.5 mm strips and dipped into PEG solution for 10 min for osmotic stress; subsequently, each plant was transferred into the lysis buffer and incubated at 28 °C on a shaker with 60 rpm for 4–6 h to release protoplasts. Protoplast were harvested with mild centrifugation and re-suspended in the MMG buffer to attain a concentration of 2 × 10^6^ cells/mL. For transfection, 100 µg plasmids were transformed into 1 mL protoplasts. After that, the transfected protoplasts were kept in the dark for 16 h at 25 °C, which was later used for RNA extraction. Total RNA was extracted from protoplasts and treated with DNase I to erase the transfected plasmids. The clean RNA samples were used to synthesize cDNA as above, ready for further qRT-PCR analysis.

### 4.11. Promoter Activation Assays

The luciferase/β-glucuronidase (LUC/GUS) reporter system was used to perform promoter activation assay. Promoter fragments of maize *ZmAAO3* genes were cloned from maize gDNA extracted from Mo17 seedlings and ligated into the vector (pBI221-*Ubi*-LUC) to replace the native *Ubi* promoter. Next, to examine the activation of the promoter by *ZmWRKY79*, the promoter-LUC (pBI221-p*AAO3*-LUC) constructs were co-transformed with the construct pBI221-*Ubi*-WRKY79 (*ZmWRKY79* transient overexpression under the control of the *Ubi* promoter) into maize leaf protoplasts. pBI221-*Ubi*-GUS (empty vector) was co-transformed as the internal control. Afterwards, protoplasts were incubated overnight and then moved into the CCLR lysis solution (1 mM EDTA, 10% glycerol, 100 mM KH2PO4 (pH 7.8), 7 mM β-mercaptoethanol, and 1% Triton X-100) for protein extraction. Luciferase assay reagent (Promega) and 4-methylumbelliferyl-β-D-glucuronide were utilized for the LUC and GUS enzymatic assay. Each assay was conducted by at least three biological replicates.

### 4.12. RNA-Seq Analysis

The aerial tissues of the WT and *ZmWRKY79* overexpression line (OE-8) were taken from the seedlings at four-week-old stages after 20% PEG6000 treatment for 24 h. A total of eight individual plants were used for each sample collection, and overall, three replications were examined. Total RNA was extracted for cDNA synthesis. The construction of the cDNA library, sequencing, and data filtering were performed by Sangon Biotech. The differentially expressed genes (DEGs) were analyzed using DESeq2 (log2 foldchanges ≥ 1) [49]. Enrichment analyses and gene ontology (GO) annotation including molecular function, cellular component, and biological process were analyzed using TopGO and the online tool agriGO (http://bioinfo.cau.edu.cn/agriGO/, accessed on 15 September 2021) [50]. Kyoto Encyclopedia of Genes and Genomes (KEGG) pathways were analyzed by clusterProfiler [51]. The RNA-seq data are listed in Supplementary Dataset 2 and 3.

### 4.13. Virus-Induced Gene Silencing (VIGS) of ZmWRKY79 in Maize

For VIGS of *ZmWRKY79*, the cucumber mosaic virus (CMV)-induced gene silencing in maize (CMV-VIGS) method was employed [52]. In short, three different constructs—pCM301, pCM201 and pCMV101—were made and transferred to *A. tumefacien*. Next, Agrobacterium culture containing all of these constructs was penetrated into the *Nicotiana benthamiana* leaves according to procedure as described previously [53]. The injected leaves of *N. benthamiana* were ground in the 0.1 M phosphate buffer and centrifuged at 3000 rpm (4 °C) for 3 min, and supernatant containing virus particles was taken and utilized for vascular puncture inoculation in maize. For vascular inoculation, about 15–16 μL supernatant were poured on an imbibed maize kernel surface (seeds were imbibed in water for 40 min in advance) and also incorporated into the scutellum with puncture [52]. Inoculated seeds were transformed into pots after incubation for 2 d at 25 °C. GFP was used as the negative control. The maize seeds with VIGS were germinated in soil and grown until the three-leaf stage for natural drought treatment as above. The silence efficiency was determined by qRT-PCR analysis at the 7th day of drought treatment. After 12 d drought treatment, roots were observed and measured to determine root length and root width, and leaves were collected for DAB staining. Survival rates were calculated after 3 d rewatering.

### 4.14. Phylogenetic Analysis of Maize WRKY Transcription Factor Family

The sequences of maize WRKY family transcription factor were downloaded from MaizeGDB (archive.maizegdb.org, accessed on 15 September 2021). The CLC Sequence Viewer 7.0 (CLC bio) software was used for phylogenetic analysis by the neighbor-joining method, and the gene information of WRKY TFs is listed in Table S2. The sequence identity was analyzed using DNAMAN software.

## Data Availability

Not applicable.

## Supplementary Materials

The following are available online at https://www.mdpi.com/article/10.3390/ijms221810080/s1.
