# Supplementary material for: Maize WRKY Transcription Factor ZmWRKY79 Positively Regulates Drought Tolerance through Elevating ABA Biosynthesis
Source: Int J Mol Sci. 2021 Sep 18;22(18):10080. doi: 10.3390/ijms221810080 (PMC8468953; doi:10.3390/ijms221810080)
Supplement: Supplementary file 1 [file ijms-22-10080-s001.zip › ijms-1385078-SI/SI-W79/SI-W79.pdf]

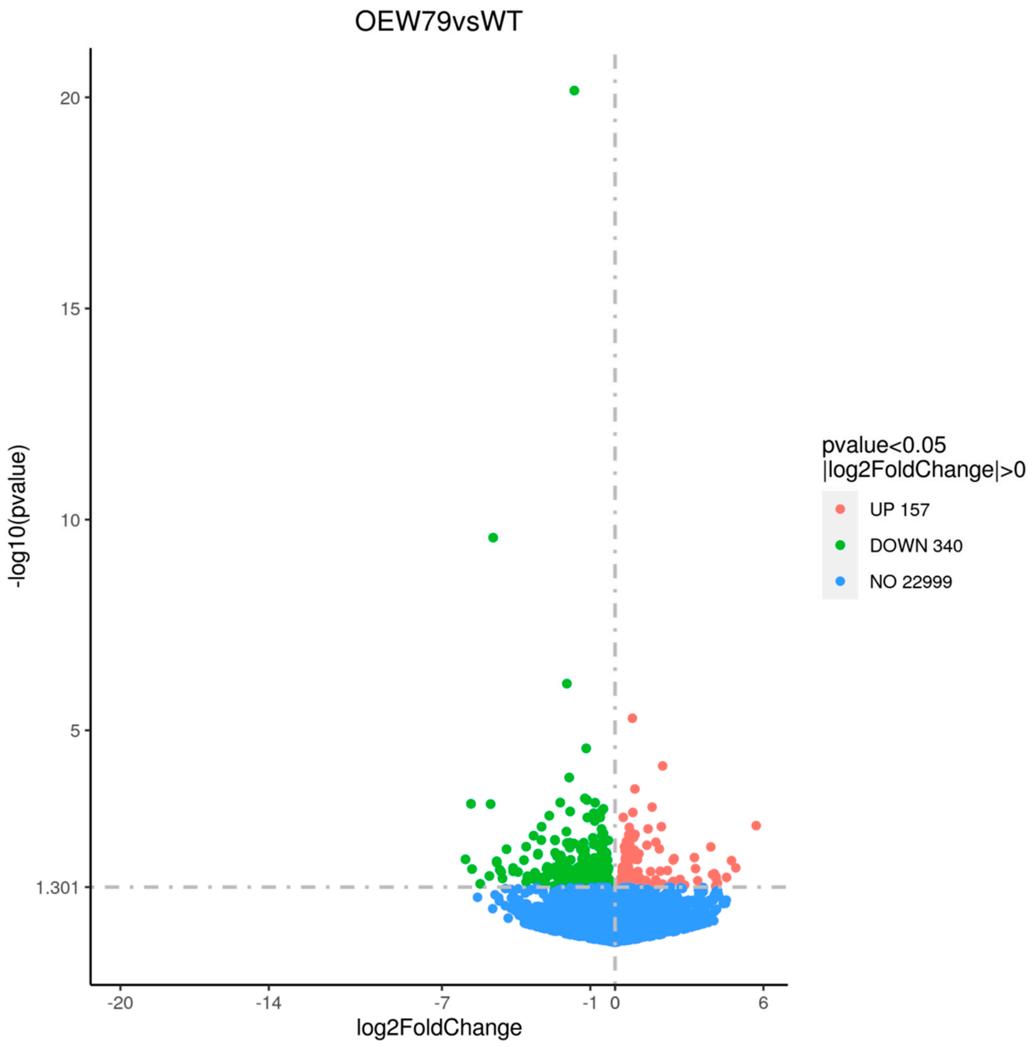

Figure S1: Volcano Plot of DEGs in ZmWRKY79-OE plants compared to WT under drought stress. Upregulated genes are indicated by red dot, down regulated genes are indicated by green dots and non-differentiated genes are indicated by blue dot. X axis signifies log2 (Fold change) and Y axis signifies -log 10(p value).

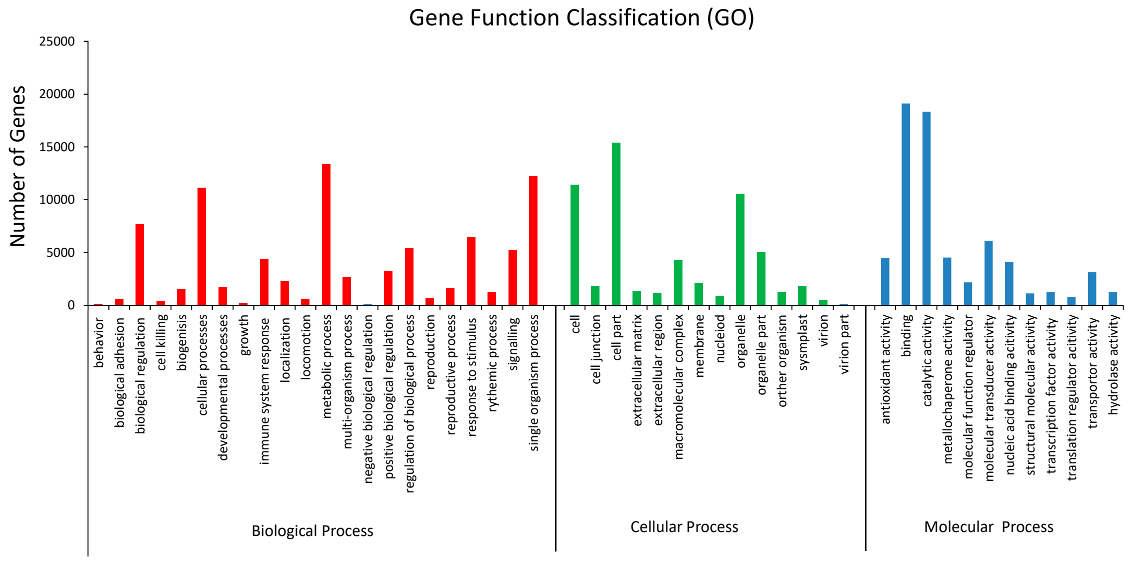

Figure S2: Gene ontology (GO) enrichment analysis of DEGs in WT and ZmWRKY79-OE plants under drought stress. Classification of up and down regulated differentially expressed gens in ZmWRKY79-OE and WT plants. Y axis represents number of genes while X axis represents the name of categories.

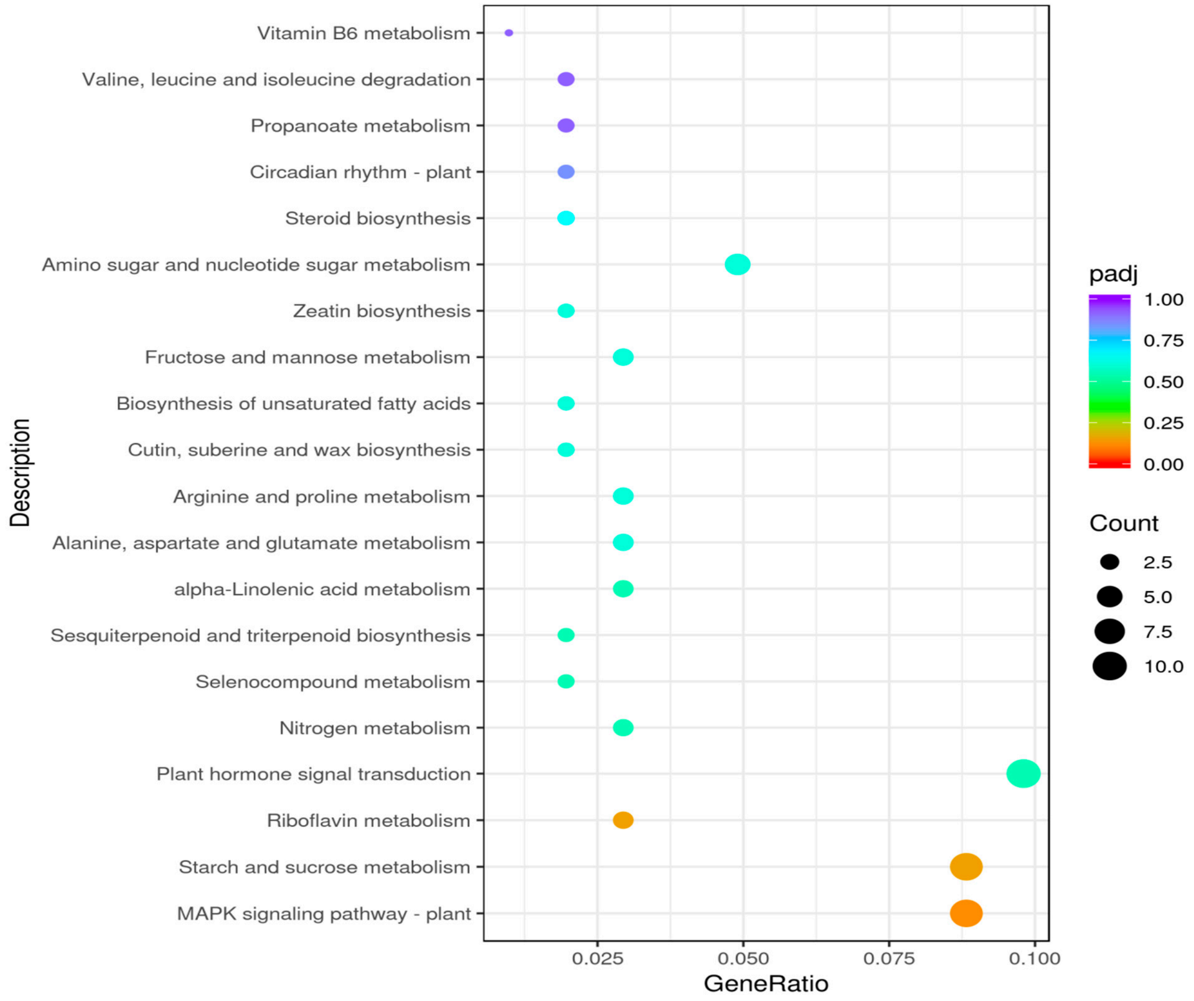

Figure S3. KEGG enrichment of the DEGs in ZmWRKY79-OE leaves compared to WT under drought stress. Y axis indicates the name of pathways while X axis indicates the rich factor. Color specifies the q value and size of dots specify the number of genes.

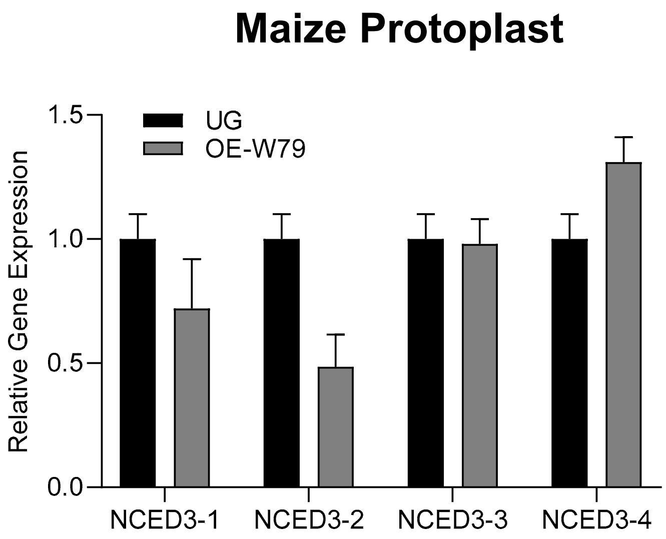

Figure S4: Expression analysis of *ZmNCED3* genes in maize protoplast with ZmWRKY79 transient over-expressed. *Ef1a* was used as the endogenous control and UG indicates the empty vector control. Error bars represent standard deviation among replication (n=3).

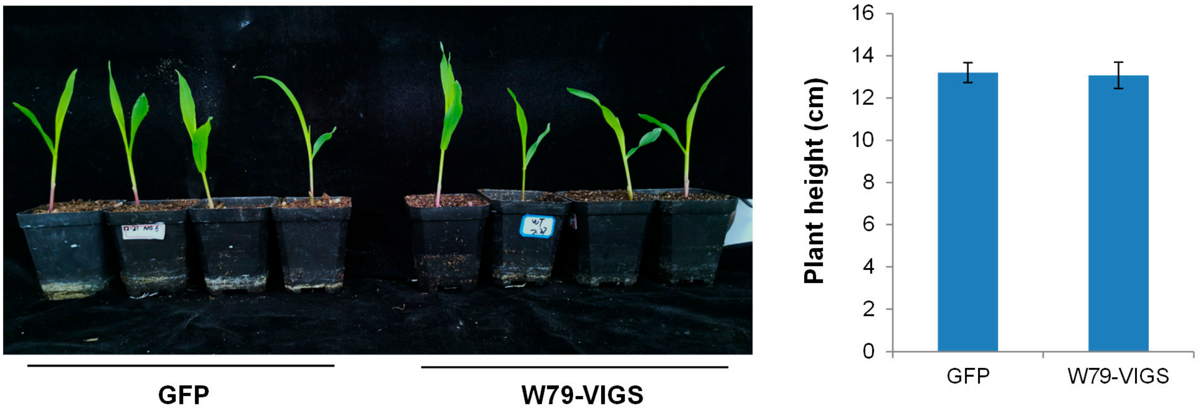

Figure S5. The growth of W79-VIGS and GFP plants under normal condition. The picture was taken at two leaves stage before drought treatment. The height of each plant was measured. Error bars indicate SE (n=4).

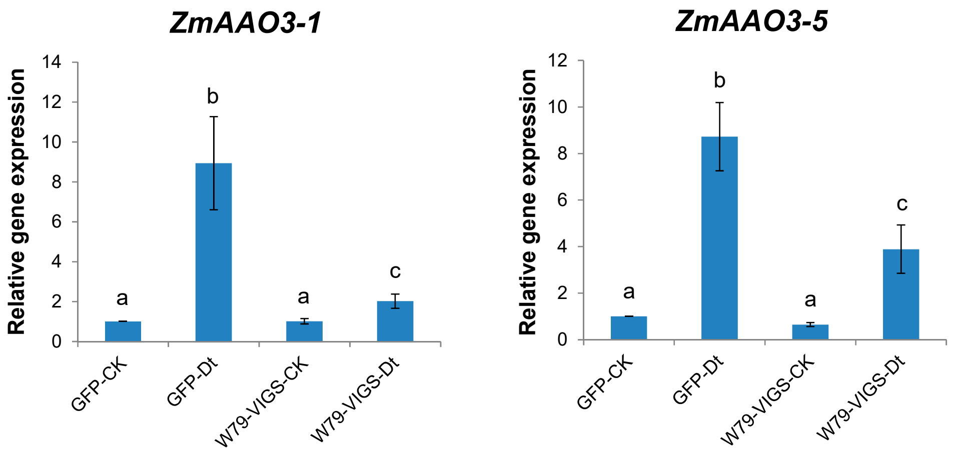

Figure S6. Expression analysis of *ZmAAO3-1* and *ZmAAO3-5* in W79-VIGS plants under drought stress. q-PCR analysis of the expression level of *ZmAAO3-1* and *ZmAAO3-5* in GFP and W79-VIGS maize leaves under normal condition (CK) or after 7 days' natural drought treatment (Dt). *Ef1a* was used as the endogenous control. Different lowercase letters indicate significant difference (LSD test, *P<0.05*). Error bars indicate SE (n=3).

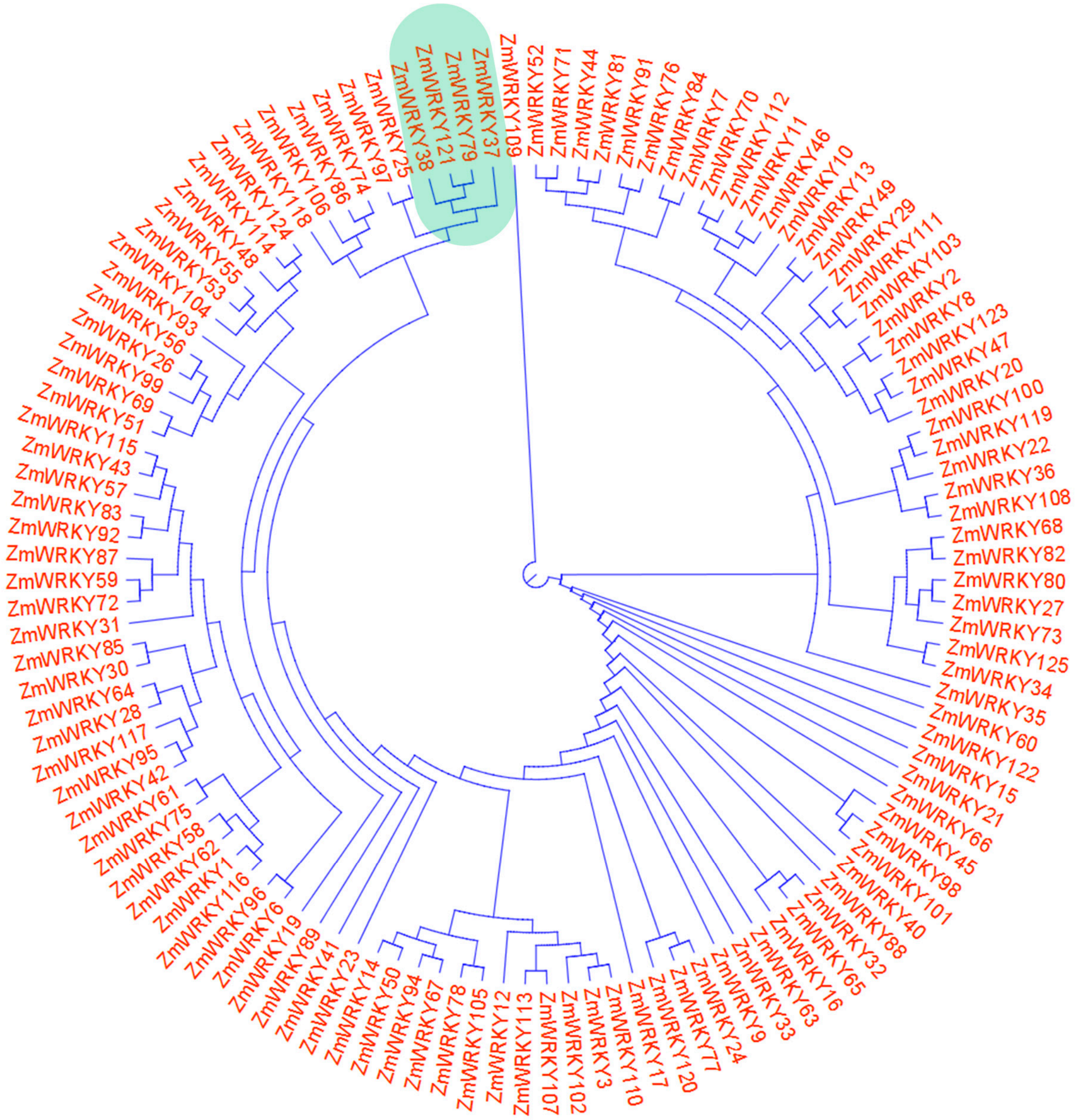

Figure S7. Phylogenetic analysis of maize WRKY transcription factor family. CLC Sequence Viewer 7.0 (CLC bio) software was used to construct this phylogenetic tree with neighbor joining method. The green oval indicated most related genes to ZmWRKY79.

Table S1: Primers used in this study

Name	**Primer sequence (5’-3’)**
ZmWRKY79	F:GACATAGATCTAGACGACATCGC
	R:CGGTCACATACTACGCCACA
ZmEf1α	F: AGGCTGACTGTGCTGTCCTT
	R: ACGTGCCTTGGAGTATTTGG
AtRD29A	F: AAGCAATGAGCATGAGCAAG
	R:GGAAGACACGACAGGAAACAC
ABI5	F:ATTGGCGGAGTTGGAGAGGAAGAG
	R:TCGGTTGTGCCCTTGACTTCAAA
AtAct	F: GTCTGGATTGGAGGGTC
	R:TGAGAAATGGTCGGAAA
AtNCED3	F: ACAGCCTCGTCCCTAAGTCT
	R: GCCCTCCCTCCTAAAGTGAC
AtAAO3	F: TCCATCATGGACTGCTCCTTC
	R: CGAGACACTAGCGCCAAGAAA
AtAOC2	F:ATGTTCGGTCTCGGAGATCTCGT
	R:GCGAGGAACGAATCCTCGTA
AtOPR2	F:ATGCGATTCGAGCTGGTTTCG
	R:CTACACCTTCCACTACTTG
AtRD22	F:ACTTGGTAAATATCACGTCAGGGCT
	R: CTGAGGTGTTCTTGTGGCATACC
AtCAT3	F: GACTACATGTCCCACTTGCC
	R: TCTTGATCCCACAAGTTGGT
AtPOD	F: CGTGCCCTTCATATTGT TGG
	R: GACGCCATCAACAAC GAGTC
ZmAAO3-1	F: CCTTGGCATTCCATTTCACA
	R:GCAACATTGGGCTCACATCC
ZmAAO3-2	F: CGCACTAGACACTAACACCG
	R:TTGGTCGAAGATTAACCTCATA
ZmAAO3-3	F: GTTGCACCGTCCTGTCCGTAT
	R:CCGCATCGCAGACTTTGTAG
ZmAAO3-4	F: TGAAGCACCCACATACACGC
	R:GCCCGAAGACTCACCTCATA
ZmAAO3-5	F: CTTTGAGACTTTCCGAGCAG
	R:AGGGAGGATAGGAAGGTAAACA
Primers for promoter cloning	
ZmAAO3-1	F:ACCGAGTGCCAAAATAAGACACT
	R: GCCTCGCATGCACTCACAGACGA
ZmAAO3-5	F:ACTATCGAGTCAGCGACGGTCGATCCC

	R: ATCTCCATCCTCTAGCAGGTA
Primers for subclone promoters	
ZmAAO3-1- 221	F: ACGCCAAGCTTGCATGCCTGCAGACCGAGTGCCAAAA
	R: CTTTGCACTTGGATCCTCTAGAGCCTCGCATGCACT
ZmAAO3-5- 221	F: ACGCCAAGCTTGCATGCCTGCAGACTATCGAGTCAGC
	R: CTTTGCACTTGGATCCTCTAGAATCTCCATCCTCTA
Primers used for W-box mutation	
AAO3-1M	F:AGACGGATATCGAATCTTAAAAATTGGCGCGAGCAACTAGGC
	R:GCCTAGTTGCTCGCGCCAATTTTTAAAGATTCGATATCCGTCT
AAO3-5M1	F:CATCACACTGATACTGCCCAAAAAATGCACAGTGTGCGT
	R:ACGCACACTGTGCATTTTTTGGGCAGTATCAGTGTGATG
AAO3-5M2	F:CTAGCACAATTAGGAGAATAAAACGACACATAGGCGTTCCGT
	R:ACGGAACGCCTATGTGTCGTTTTATTCTCCTAATTGTGCTAG

Table S2: Gene information of WRKY transcription factors in maize for phylogenetic analysis.

Name	Gene ID	Chr	SubGr.	Name	Gene ID	Chr	SubGr.
**ZmWRKY32**	GRMZM2G030272	1	III	ZmWRKY48	GRMZM2G120320	5	IIa
**ZmWRKY75**	GRMZM2G425430	1	I	ZmWRKY2	GRMZM2G048450	5	IIe
**ZmWRKY60**	GRMZM2G383594	1	IIe	ZmWRKY78	GRMZM2G073272	5	IId
**ZmWRKY3**	GRMZM2G130374	1	IId	ZmWRKY46	GRMZM5G823157	5	IIe
**ZmWRKY25**	GRMZM2G324999	1	III	ZmWRKY62	GRMZM2G143765	6	I
**ZmWRKY102**	GRMZM2G070211	1	IId	ZmWRKY114	AC209050.3_FGP003	6	IIa
**ZmWRKY96**	GRMZM2G149219	1	IIc	ZmWRKY13	GRMZM2G156529	6	IIe
**ZmWRKY107**	GRMZM2G018487	1	IId	ZmWRKY125	GRMZM2G163054	6	IIc
**ZmWRKY30**	GRMZM2G143204	1	IIc	ZmWRKY21	GRMZM2G127064	6	III
**ZmWRKY31**	GRMZM2G008029	1	I	ZmWRKY83	GRMZM2G012724	6	I
**ZmWRKY12**	GRMZM2G164082	1	IId	ZmWRKY57	GRMZM2G169966	6	I
**ZmWRKY10**	GRMZM2G083717	1	IIe	ZmWRKY100	GRMZM2G401521	6	IIc
**ZmWRKY50**	GRMZM2G071907	2	IId	ZmWRKY83	GRMZM5G863420	6	IIc
**ZmWRKY70**	GRMZM2G024898	2	IIe	ZmWRKY117	GRMZM2G453571	6	IIc
**ZmWRKY101**	GRMZM2G123387	2	IIc	ZmWRKY51	GRMZM2G366795	6	IIb
**ZmWRKY72**	GRMZM5G816457	2	I	ZmWRKY85	GRMZM2G018721	7	IIc
**ZmWRKY105**	GRMZM2G102583	2	IId	ZmWRKY37	GRMZM2G139815	7	III
**ZmWRKY23**	GRMZM2G400559	2	III	ZmWRKY79	GRMZM2G025895	7	III
**ZmWRKY24**	GRMZM2G099593	2	III	ZmWRKY53	GRMZM2G125653	7	IIa
**ZmWRKY38**	GRMZM2G163418	2	III	ZmWRKY104	GRMZM2G169149	7	IIa
**ZmWRKY55**	GRMZM2G057011	2	IIa	ZmWRKY52	GRMZM2G151407	7	I
**ZmWRKY71**	GRMZM2G052671	2	I	ZmWRKY1.1	GRMZM2G398506	7	I
**ZmWRKY116**	GRMZM2G130854	2	I	ZmWRKY17	GRMZM2G381378	7	III
**ZmWRKY108**	GRMZM2G106560	2	IIc	ZmWRKY49	GRMZM2G006497	8	IIe
**ZmWRKY93**	GRMZM5G871347	3	IIb	ZmWRKY80	GRMZM2G516301	8	IIc
**ZmWRKY27**	GRMZM2G475984	3	IIc	ZmWRKY26	GRMZM2G083350	8	IIb
**ZmWRKY56**	GRMZM2G176489	3	IIb	ZmWRKY69	GRMZM2G448605	8	IIb
**ZmWRKY45**	GRMZM2G151444	3	IIc	ZmWRKY106	GRMZM2G063880	8	III
**ZmWRKY99**	GRMZM2G327349	3	IIb	ZmWRKY20	GRMZM2G414315	8	IIe
**ZmWRKY110**	GRMZM2G173680	3	IId	ZmWRKY34	GRMZM2G057116	8	IIc
**ZmWRKY58**	GRMZM2G076657	3	I	ZmWRKY121	GRMZM2G061408	8	III
**ZmWRKY120**	AC165171.2_FGP002	3	IIc	ZmWRKY92	GRMZM2G449681	8	I
**ZmWRKY43**	GRMZM2G148087	3	I	ZmWRKY89	GRMZM2G034421	8	III
**ZmWRKY81**	GRMZM2G059562	3	III	ZmWRKY44	GRMZM2G432583	8	III
**ZmWRKY40**	GRMZM2G158328	3	III	ZmWRKY119	GRMZM2G015433	8	IIc
**ZmWRKY41**	GRMZM2G065290	3	III	ZmWRKY68	GRMZM2G137802	8	IIc
**ZmWRKY19**	GRMZM2G382350	3	III	ZmWRKY84	GRMZM2G304573	8	IIb
**ZmWRKY103**	GRMZM2G141299	3	IIe	ZmWRKY47	GRMZM2G149683	8	IIe
**ZmWRKY29**	GRMZM2G040298	3	IIe	ZmWRKY28	GRMZM2G145554	8	IIc
**ZmWRKY73**	GRMZM2G101405	3	IIc	ZmWRKY86	GRMZM2G411766	8	III
**ZmWRKY95**	GRMZM2G151763	3	IIc	ZmWRKY42	GRMZM5G812272	8	IIc
**ZmWRKY74**	GRMZM2G408462	3	III	ZmWRKY66	GRMZM2G045560	8	IIc

**ZmWRKY63**	AC198725.4_FGP009	3	IIc	ZmWRKY115	GRMZM2G036703	8	I
**ZmWRKY123**	GRMZM2G105140	3	IIe	ZmWRKY88	GRMZM2G029282	8	III
**ZmWRKY7**	GRMZM2G354384	3	IIb	ZmWRKY35	GRMZM2G029292	8	III
**ZmWRKY36**	GRMZM2G054125	4	IIc	ZmWRKY111	GRMZM2G013391	8	IIe
**ZmWRKY122**	GRMZM2G549512	4	I	ZmWRKY22	GRMZM2G111354	8	IIc
**ZmWRKY94**	GRMZM2G148561	4	IId	ZmWRKY118	GRMZM2G003551	9	III
**ZmWRKY97**	AC205562.3_FGP002	4	III	ZmWRKY124	GRMZM2G111711	9	IIa
**ZmWRKY98**	GRMZM2G377217	4	IIc	ZmWRKY61	GRMZM2G171428	9	I
**ZmWRKY11**	GRMZM2G138683	4	IIe	ZmWRKY65	GRMZM2G060918	9	III
**ZmWRKY16**	GRMZM2G063216	4	III	ZmWRKY63	GRMZM2G005207	10	III
**ZmWRKY76**	GRMZM2G451035	4	III	ZmWRKY91	GRMZM2G441031	10	III
**ZmWRKY77**	GRMZM2G461648	4	III	ZmWRKY67	GRMZM2G090594	10	IId
**ZmWRKY9**	GRMZM2G169564	4	III	ZmWRKY15	GRMZM2G004060	10	III
**ZmWRKY87**	GRMZM2G027972	4	I	ZmWRKY33	GRMZM2G020254	10	I
**ZmWRKY8**	GRMZM2G038158	4	IIe	ZmWRKY59	GRMZM2G031963	10	I
**ZmWRKY113**	GRMZM2G147880	5	IId	ZmWRKY112	AC208110.2_FGP001	10	IIe
**ZmWRKY6**	GRMZM2G161411	5	IIc	ZmWRKY14	GRMZM2G091331	10	IId
**ZmWRKY109**	GRMZM5G880069	5	IId				
